# An Ambient Intelligence Framework for End-User Service Provisioning in a Hospital Pharmacy: a Case Study

**DOI:** 10.1007/s10916-015-0298-z

**Published:** 2015-08-19

**Authors:** Diego Martín, Ramón Alcarria, Álvaro Sánchez-Picot, Tomás Robles

**Affiliations:** Technical University of Madrid, Av. Complutense 30, 28040 Madrid, Spain

**Keywords:** End-user development, Case study, Self-care, Active aging, ECA rules, Drug traceability, Hospital pharmacy, RFID

## Abstract

End-user development is a new trend to provide tailored services to dynamic environments such as hospitals. These services not only facilitate daily work for pharmacy personnel but also improve self-care in elder people that are still related to hospital, such as discharged patients. This paper presents an ambient intelligence (AmI) environment for End-user service provisioning in the pharmacy department of Gregorio Marañón Hospital in Madrid, composed of a drug traceability infrastructure (DP-TraIN) and a ubiquitous application for enabling the pharmacy staff to create and execute their own services for facilitating drug management and dispensing. The authors carried out a case study with various experiments where different roles from the pharmacy department of Gregorio Marañón Hospital were involved in activities such as drug identification, dispensing and medication administering. The authors analyzed the effort required to create services by pharmacy staff, the discharged patients’ perception of the AmI environment and the quantifiable benefits in reducing patient waiting time for drug dispensing.

## Introduction

The development of applications for AmI environments usually involves complex systems and high experienced developers, which incurs in high efforts and costs. User involvement in co-creation processes [1] is a new trend to provide applications and services particularized more efficiently to problems only understood by domain experts, who often do not have computer science or programming skills to create such applications or services. End-user development (EUD) refers to activities and tools to allow end-users to develop their own programs, satisfying their new requirements and saving efforts and costs. These users are also called prosumers [2]. They were originally producers and consumers of content but they are recently motivated to add new functionality to their tools, platforms and AmI environments.

Current processes for drug dispensing are complex [3–5] in nature and require a moderate interaction between the patient and the hospital pharmacy, which means more time spent, queues, and sometimes dispensing errors. Using a framework for service development by pharmacy staff would allow a more dynamic system, adapted to the hospital infrastructure.

The authors carried out an experimental validations in order to validate the AmI environment presented in this paper and to address the next three research questions that guided this research work:

- RQ1: Can the hospital pharmacy staff develop services using an AmI environment application?
- RQ2: What is the discharged patients’ perception of an AmI environment application?
- RQ3: Can an AmI environment reduce the waiting time for patients when receiving their medicines?

The experimental validation was divided in three different experiments, one per research question, where different roles from the pharmacy department of Gregorio Marañón Hospital were involved as follows:

- RQ1: 25 people from the pharmacy staff and interns created four services by themselves using the AmI environment.
- RQ2: 28 discharged elder patients used a self-care ubiquitous application for drug monitoring.
- RQ3: 98 patients with prescribed medicines used a specific service provided by the AmI environment designed to reduce the waiting time for drug dispensing.

The evidence and findings obtained during the experimental validation are very enlightening and indicate that an AmI environment is a reliable solution in a hospital pharmacy where the pharmacy staff is able to develop simple services to facilitate daily tasks and discharged elder patients can be involved and helped.

This paper is an extended version of our previous work [6], which proposed an infrastructure for tracing drugs at the pharmacy based on RFID technology and a prosumer platform designed to create and provisioning services in the pharmacy. This paper proposes a complete validation and experimentation where 151 participants were involved in three different experiments in order to answer the three research questions analyzed in this research work.

## Related work

This section describes related work studying EUD techniques applied to hospital environments.

There are studies that explored the characteristics of frameworks [7] enabling the creation of prosumer services [8]. These tools offer different creation strategies [1], such as natural languages and, semi-automatic solutions requiring more effort from the users [9]. In our approach we combine the benefits of the mashup tools and the adaptation of the ECA (Event, Condition, Action) rules concept [10]. Also we measure user satisfaction with prosumer environments, considering the effort required to create prosumer services using an AmI environment. There are few studies analyzing this aspects.

Service creation for end-users produces several benefits for hospital environments. According to the Attention Investment model [11] which offers a cost/benefit analysis that predicts the circumstances in which end-users will choose to engage in programming activities, an employee weighs four factors before taking an action, for example, before adopting a traceability system to detect patient arrival and improve drug dispensing:

This expert might expect a *pay*-*off* in that this system would allow him to detect discharged patient arrival and improve patient waiting time. As a *perceived cost*, he will have to spend time learning to use the new features, and, as *perceived risk*, the system might not be flexible enough to support the variety of business cases present in hospital environments. The *perceived investment*, measured as number of attention units expended toward a potential reward, often dissuades pharmacy workers to engage in these kind of innovations. In fact, some techniques [12, 13] have explored these possibilities and define a trade-off point by which the project is viable from the customer’s point of view.

Through the application of EUD to the medical domain we can maintain low the perceived risk, as a service development platform is flexible enough to create services adapted to changing situations in the medicine dispensation process. In our work we focus on minimizing the perceived investment by validating that our proposed service development platform is easy enough to be used by non-experts in computer science and with no programming skills (related to RQ1). We also seek to increase the expected pay-off, as services created by this platform improves drug dispensing and medication administering processes (related to RQ2 and RQ3).

The application of EUD to the medical domain [14] is less explored. The work of Sadasivam and Tanik [15] describes the development of a solution that allows the possibility to compose and recompose domain specialist tools to adapt to continually changing needs. The study of Morrison and Blackwell [5] describes four experiences related to the application of EUD techniques in the customization of Electronic Patient Records.

In the field of healthcare, as far as we know there is not any work related the concepts of EUD to hospital services. There are some works centered on drug identification and their interactions [16] and some works have focused on reducing patient wait times to improve resource utilization and increase hospital service quality. To do that they apply different techniques such as simulation [17] and other mathematical models to provide better decision-making strategies [18].

## Drug management and dispensing scenario

The service development platform is designed to cover various situations related to patient interaction with the hospital pharmacy. We have modelled these situations in four use cases to facilitate drug management and dispensation, reducing human interaction and the complexity of some tasks.

We consider a hospital pharmacy incorporating an infrastructure to register and identify drugs via RFID tags when they cross certain corridors with RFID antennas installed. The implications, benefits and procedure to incorporate a traceability infrastructure into a hospital pharmacy department is out of the scope of this paper and it is also described in our previous work [1, 6].

The first use case (Use Case #1) to be implemented into our service platform is *Drug identification and storage*: Drugs are tagged by RFID and associated to a unique identifier. Since in the hospital pharmacy there are many entry points for medicines (from the laboratory in the case of compounded medication, from the room where medicine shipments are received) and output points (to a medical unit, dispensed to discharged patients) it is needed to correctly model the transit of drugs and the stock that is kept in the pharmacy at a given time. A useful service in this case is to check if the available stock of a medication is enough and if it is below a threshold a drug order is generated through the connection to the fax.

Use Case #2 refers to *Drug dispensing to discharged patients*: After being discharged from the hospital, patients with pneumonia, congestive heart failure or myocardial infarction (MI) can typically have a dozen or more medications to take at varying times and doses. To reduce waiting time of patients the medication is bundled the pharmacy assistant once the system detects that the patient enters the hospital pharmacy building. The patient approaches to the counter, shows the prescription and the system records the dispensation process, being automatically linked to the patient ID.

Use Case #3 refers to *Medication administering assistance*: Medication schedule often changes repeatedly, as physicians review an individual’s response and side effects, and then make adjustments accordingly. Consequently, staying on the appropriate medication regimen can be difficult, at best, for a patient in a clear state of mind, but for many who struggle with multiple health problems, it can be nearly impossible for them to accurately comply with their medication schedule. A mobile application must be developed to guide the user to take the medicines.

Use Case #4 refers to *Missing a medication dose*: the drug administration infrastructure is notified by the mobile application when an individual appears to have failed to take the prescribed medication. It could be for example an e-mail indicating that patient missed a 7 p.m. dose. If the patient has a caregiver an e-mail or text message can be sent to that individual as well, letting him or her know that the patient has not taken her medication as prescribed.

## An ambient intelligence environment for drug provisioning in hospital pharmacy

**Fig. 1:**
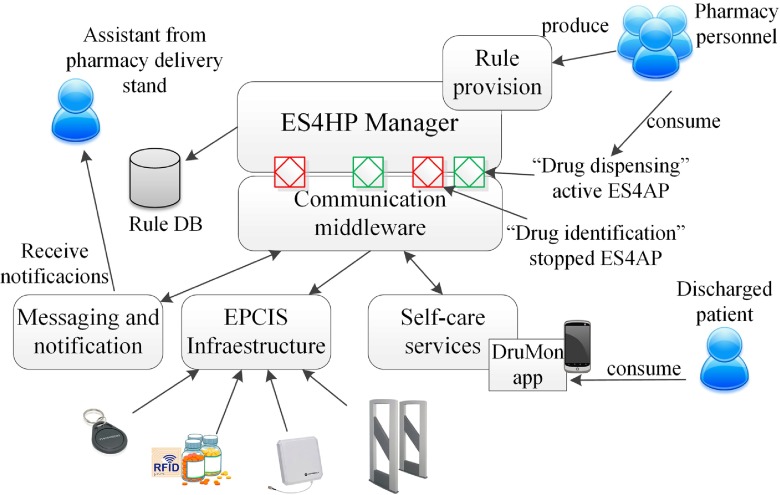
Functional architecture of the AMI solution

In this section we describe the deployment of an AmI environment composed of the following elements: DP-TraIN (Drug and patient Traceability Infrastructure), a system for drug traceability in the pharmacy department of Gregorio Marañón Hospital. ES4HP (End-user services for hospital pharmacy) a data model for describing services that works over DP-TraIN, ES4HP Manager, a tool for managing (creating and provisioning), and finally DruMon (Patient self-care drug monitor), an application that assist patients for medication administering at home. Figure 1 describes the functional architecture of the proposed solution.

### DP-TraIN

DP-TraIN is an AmI environment deployed at the hospital pharmacy department of the Hospital. The deployment was performed by an SME through a research project called UNICA ID.

DP-TraIN is an EPCIS infrastructure consisting in a drug registration and delivery stand, some RFID arches for tracing drugs located at the corridors near the pharmacy, and also an RFID arch for identifying selected discharged patients at the main entrance of the hospital.

**Fig. 2:**
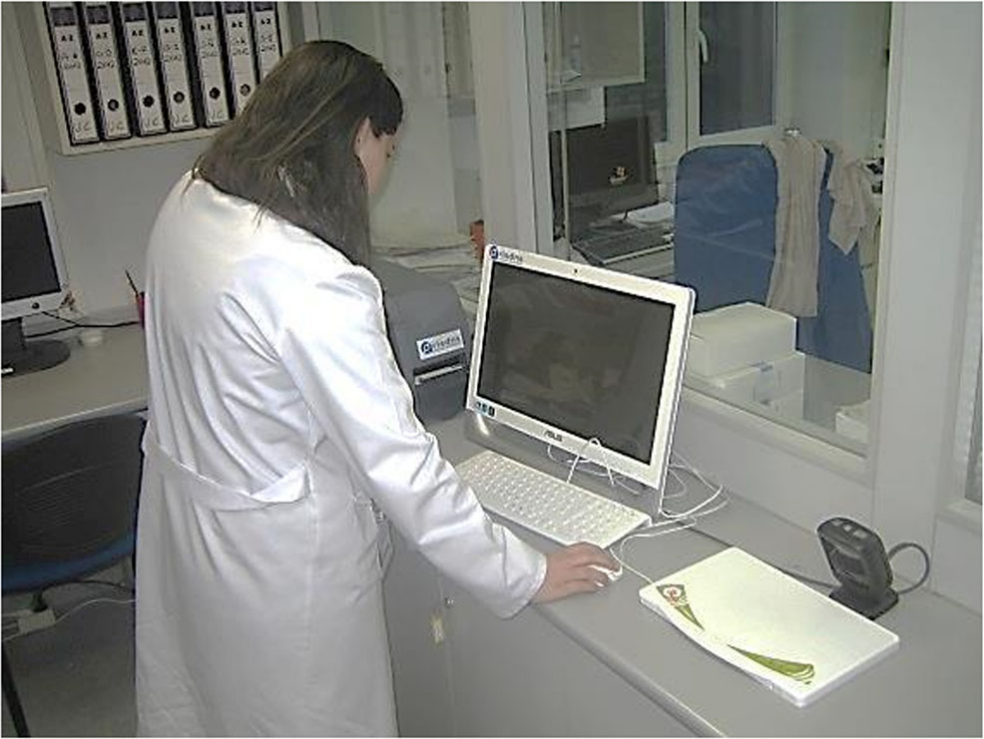
Drug registration and delivery stand

At the drug registration and delivery stand, the pharmacy staff registers the drugs using a RFID label printer. The staff can also deliver the drugs to a patient using a handheld RFID reader or a handheld barcode scanner. Figure 2 shows the deployment of this stand.

**Fig. 3:**
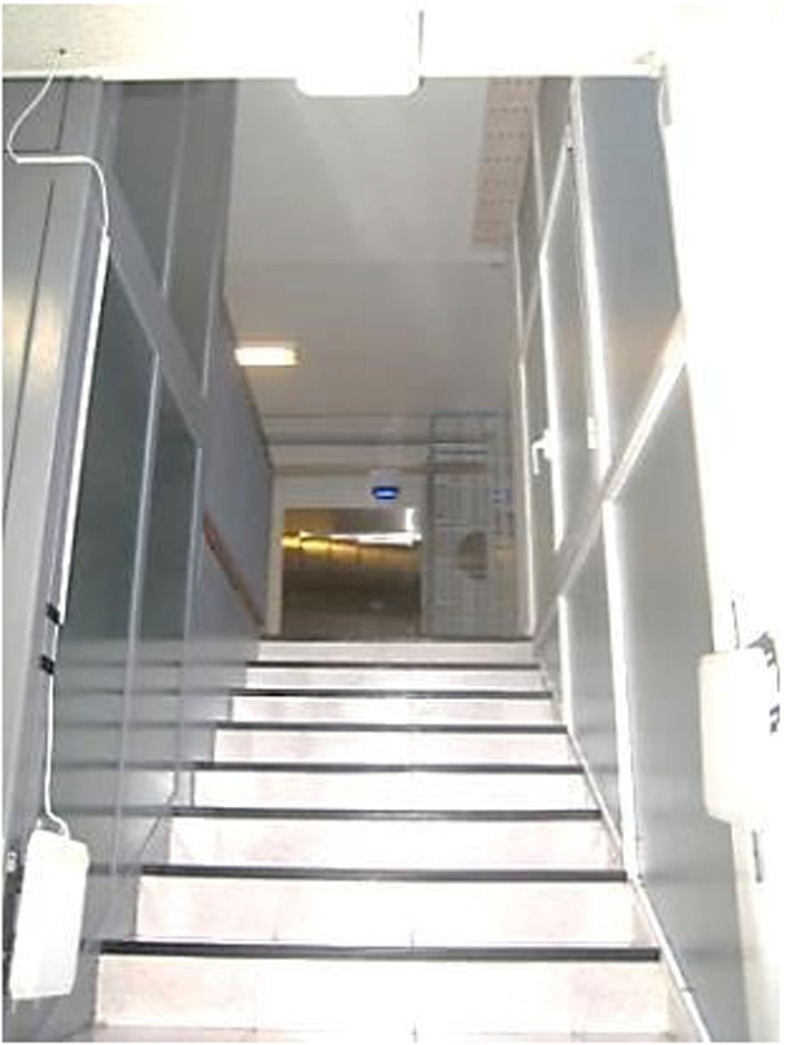
RFID arch

At the main entrance of the pharmacy four UHF antennas were disposed forming an RFID arch (as seen in Fig. 3), able to track the entry and exit of drugs.

The RFID readers and writers generate EPCIS events when they read or write an RFID tag from a drug and the system stores those events in a repository. The system also informs to ES4HP about these events.

### ES4HP manager

ES4HP Manager is an AmI tool for end-user service provisioning in a hospital pharmacy department. This tool is a full graphical tool based on cloud capabilities and executable on any device with a web browser. It is able to manage services that can run over DP-TraIN helping the pharmacy staff to trace drugs in order to add new functionality to DP-TraIN.

**Fig. 4:**
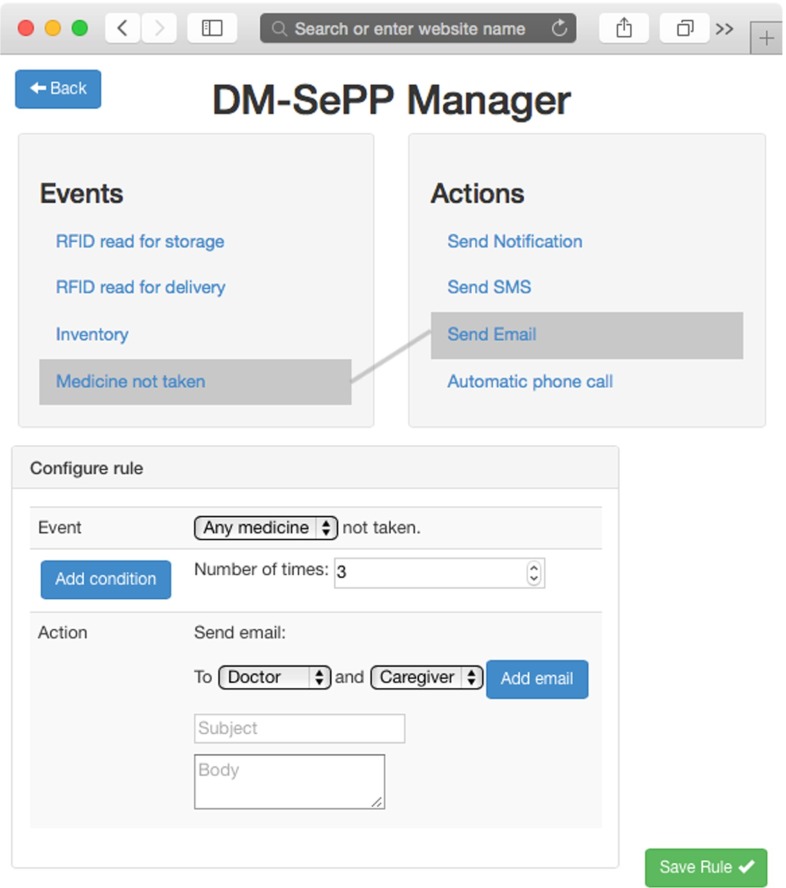
ES4HP manager creation view

**Fig. 5:**
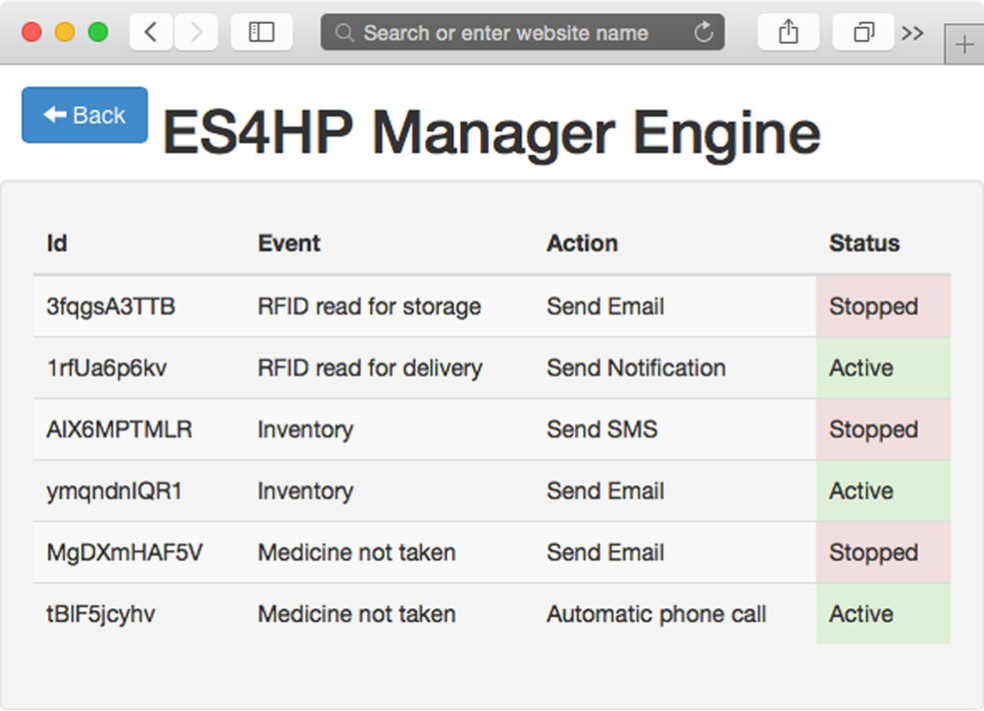
ES4HP manager - engine view

ES4HP Manager has two different operating environments: service creation and service provision environments; both are programmed using HTML5, CSS3 and JavaScript. The service creation environment helps end-users to create services through a graphical environment (see Fig. 4). The service provision environment consists of an execution engine of services where users can start, pause or stop them; the engine listens to the events produced by DP-TraIN and compares them with the events of the running services and, if they match, the engine executes the action defined in the service (as shown in Fig. 5).

The ES4HP are based on ECA (event, condition, action) rules, but in previous research [1] we showed that the “condition” element presented modelling problems for users, so in this research work this element was removed. This composition paradigm describes service composition by providing two related components: Event (E), as an occurrence triggering the action execution and action (A), describing the realization of a certain action when the event occurs.

### DruMon app

**Fig. 6:**
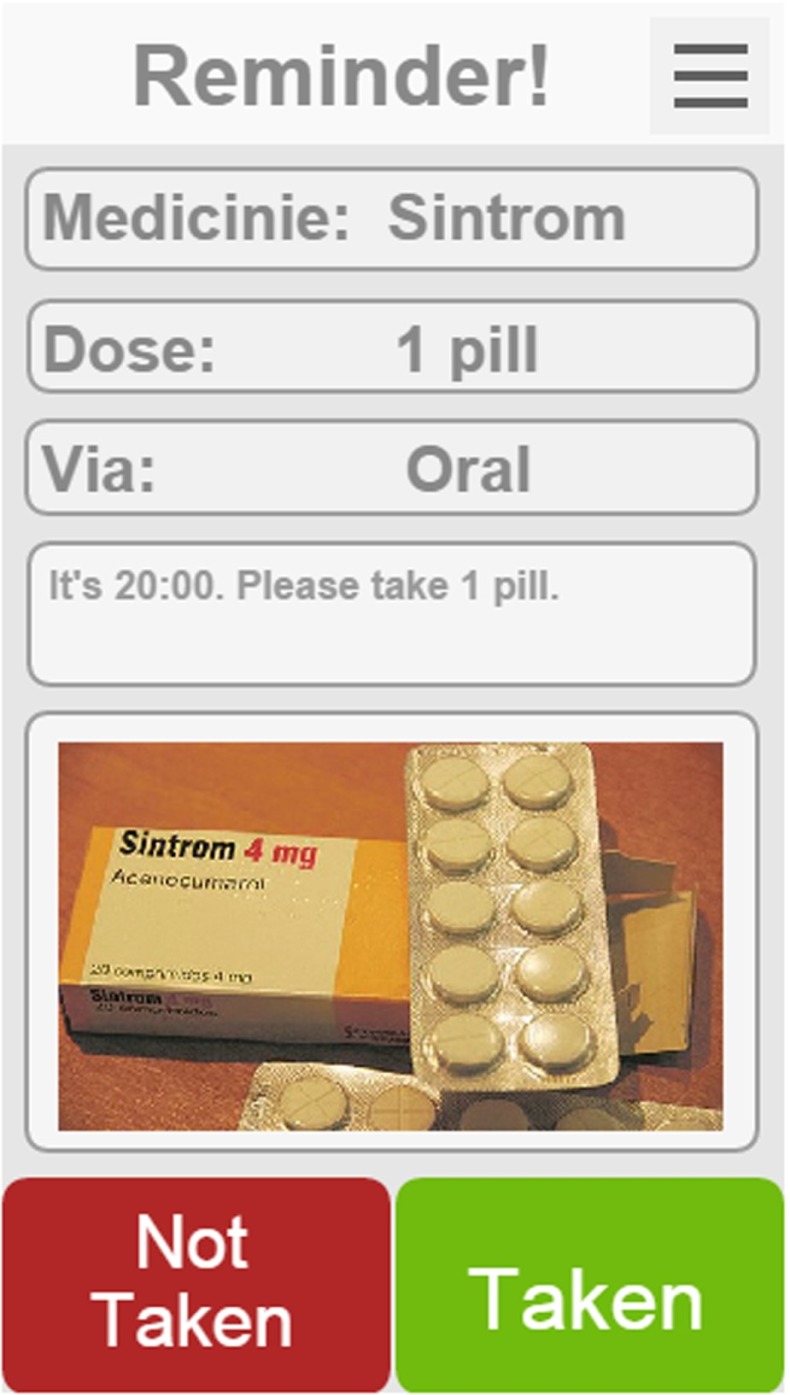
Drumon App reminder

DruMon App is a mobile application for self-care and active aging, where the patients are reminded by the system when to take the drugs, which drug, how it is administered and any other extra information. All the configuration information is obtained via Internet with the ES4HP Manager, so the reminders are configured automatically based on the physician’s information and the user only needs to login in the application. Once the alarm triggers a notification appears in the user’s device and if configured the alarm also sounds. Once the user access the application he/she has access to all the information related to the medicine as shown in Fig. 6.

The user can then inform the physician whether he/she has or has not taken the medicine. If the user ignores the alarm, after a short period the system is also informed and the process repeats some preconfigured times, the physician will be informed so that he can contact the patient or some familiar.

However, IT applications cannot replace an in-depth clinical interview, which allows medical professionals to assess the adherence to the treatment.

## Experimental validation

A case study was designed in order to analyze and respond the research questions that guided this research work. These research questions were answered using the AmI environment presented in this paper in Section 4. The case study was divided in three different experiments designed to answer each research questions; context, design and planning are different among the experiments. During this section we are going to use the research questions as a guide for each sub-section.

The authors of this research work (hereafter, experts) had little control of the people involved in the experimentations. This approach is appropriate to replicate the experiment in similar contexts. Experts guided, executed, and evaluated each experiment.

All the participants were treated anonymously by the application and the experts. No personal data were stored or diffused. Experimentations were approved by the Ethics Committee of Hospital General Universitario Gregorio Marañón in the frame of UNCA-ID project.

The experimental validation context, planning and data collection are explained for each research question.

### RQ1 - Can the hospital pharmacy staff develop services using an AmI environment application?

Twenty five people from the pharmacy staff, pharmacy interns and last year students of pharmacy (hereafter, pharmacy specialists) participated in the experimentation.

Experts selected 1 month of EPCIS records from the DP-TraIN repository, meaning more than 10.000 EPCIS events.

Pharmacy specialists, using ES4HP Manager, created four services in order to solve the four use cases described in Section 3, using concrete values for each service provided by Pharmacy Chief as example.

The case study plan was split into three phases:

- *Training phase*: Pharmacy specialists received some training in EUD, AmI philosophy, and how to configure and use the ES4HP Manager. Training sessions consisted of a 1-h lecture at the beginning of the experimental validation given by one of the experts.
- *Service Creation phase*: In this phase, the participants used ES4HP Manager to create the four services.
- *Evaluation phase*: Two experts assessed all the ES4HPs created by the Pharmacy Specialists, they evaluated specific elements of the services (shown in Table 1) to determine its quality, and then calculated their quality aggregate. The quality is collected using an 11 points Likert scale (from 0 to 10).

**Table 1:** Service quality criteria

Analyzed questions	Weight
Proper use of event element	1
Proper use of action element	1
Has the services passed the test?	1
Does the services generate precise messages?	1

Some of the services obtained a qualification of zero in each of the criteria analyzed (shown in Table 3 in Results section). They were removed from the statistical analysis in order to avoid outliers; however they are discussed in results subsection.

### RQ2 - What is the discharged patients’ perception of an AmI environment application?

Twenty eight discharged patients (hereafter, patients) volunteered to participate in the experiments; all of them are over 60 years old. They borrowed a mobile device provided by the experts during small workshop in the hospital, in order to test DruMon app presented in Section 4.3. Events containing the medication to take at the appropriate time were simulated for this workshop. The application receives a message from the ES4HP engine when the patients have to take their medication according to Use Case #3. Patients used the application for half an’ and then they were interviewed by the experts asking them about the use of the application and their perceptions.

This case study was divided in three different phases:

- *Training phase*: Each group received individualized explanation about the use of DruMon app. It is a really simple application where the patient only have to press the “Taken” button if he/she took the medicine or press “Not taken” button if not.
- *Medicine monitoring phase*: Every patient received a smartphone or tablet in order to test the application. They received simulated reminders from the application when they should take the medicine.
- *Interview phase*: The experts interviewed all patients asking them aspects and perceptions of the DruMon app. They were asked about a specific subset of metrics for Ubiquitous Computing proposed by Scholtz [19] shown in Table 2. Answers were collected in a 6-steps Likert scale (from 0 to 5).

**Table 2:** AmI environment perception criteria

Metric	Question
Focus	Is the technology appropriate?
Overhead	Does the important information is easily accessible?
Value	Does the application provides any benefit?
User satisfaction	Are you satisfied with the use of the application?
Frustration	Was the use of the application frustrating?
Utility	Do you think the application is useful?

### RQ3 - Can an AmI environment reduce the waiting time for patients when receiving their medicines?

The waiting time of 96 people with prescriptions at the pharmacy drug delivery stand was measured by the experts. The experts distributed 50 RFID cards to volunteered patients that planned to take their medication from the hospital pharmacy. The experts recorded the prescription associated to each RFID card according the Use Case #2. Thus, when a patient with an RFID card go through the hospital main entrance equipped with a RFID arch reader (as shown in Fig. 3), the arch generates an EPCIS event triggering a message at the pharmacy drug delivery stand informing that someone is coming close to the pharmacy stand with an specific prescription; allowing the personal at delivery stand to prepare the medicines while the patient is coming.

This experimentation was planned in three phases:

- *Patient selection*: A group of 50 patients with prescriptions was selected with the help of a physician associated to the internal medicine department.
- *Waiting time measurement*: Experts measured time waited by the patients while the pharmacist prepared the medicines to them.
- *Results analysis*: Experts analyzed the data obtained analyzing if the waiting time is reduced. Experts used a chronograph to take the measures since a person gives the prescription to the pharmacist until he/she leaves the stand. As said before, no waiting time in the queue was measured due to its randomness. All measurements were taken in seconds.

## Results

This section presents and discusses the results obtained in the experimental validation, according to the research questions proposed in this work.

### Analysis of the quality of the services created by the pharmacy specialists

A study about the quality of the services created by pharmacy specialists was carried out. Experts analyzed each service created by the participants. These services solve four typical use cases from the hospital pharmacy, as described in Section 3.

Table 3 summarizes the average quality of the services built by pharmacy specialists. Quality was evaluated using the criteria presented in Table 1.

**Table 3:** Service quality summary

	Use case #1		Use case #2		Use case #3		Use case #4		Total	
	Mean	SD	Mean	SD	Mean	SD	Mean	SD	Mean	SD
Proper use of event element	6.44	3.2	7.66	2.89	3.7	3.57	5.26	3.1	5.76	3.47
Proper use of action element	5.06	2.66	6.64	2.82	2.62	2.63	3.7	2.05	4.5	2.94
Test results	4.2	2.66	5.56	2.53	1.2	1.01	2.68	2.78	3.41	2.84
Quality of the messages	6.54	3.44	8.42	2.7	1.42	1.18	2.36	1.7	4.68	3.76
Quality aggregate	5.56	2.86	7.07	2.54	2.23	1.93	3.5	2.05	4.59	2.99
Number of zeros	4		2		9		4		19	

Obtained results indicate that the quality of the services created were quite good but depended on the complexity of use cases. Services created for Use case #1 and Use Case #2 obtained high quality scores for the quality aggregate (5.56 and 7.07 respectively); however services for Use Cases #3 and #4 scored considerably lower values for the quality (2.23 and 3.5 respectively).

The questions analyzed that obtained the best scores for quality were those that measured the proper use of the *Event* element followed by the proper use of the *Action* element; the reason for these good scores was that the set of possible *Events* and *Actions* are quite small and also they are well explained. The variable “Quality of the message” obtained different values depending of the Use Cases; Use Case #1 and #2 obtained better results than Use Case #3 and #4. Messages are intended to be read by humans; in the case of Use Cases #1 and #2 messages were a simple phrase; while in Use Cases # 3 and # 4 the messages required the value of a property or an object, such as *medication*, # *of missed doses*, etc., the pharmacy specialists were confused in message composition.

The question analyzed with worsts cores were the Test Results; all the services were tested by experts in order to check whether they work properly. Results showed that almost no service performed its function as expected. ES4HP Manager does not include test environment to test the services created with example data; so, the pharmacy specialists could not test the services they created in a test environment (e.g., a sandbox). It would be very interesting that EUD environments had a test environment with realistic context to improve service quality, as we discuss in Conclusions.

**Fig. 7:**
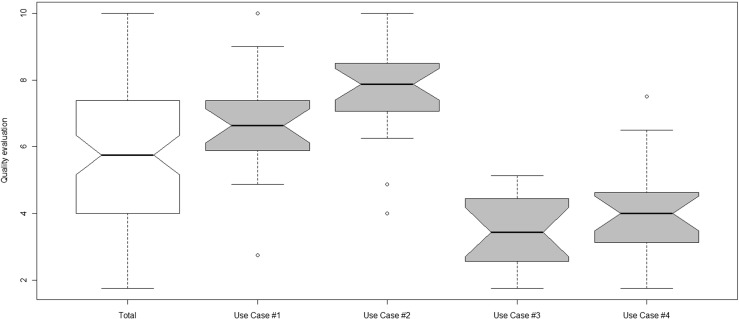
Quality distribution of the services created

Figure 7 shows the distributions of the quality scores for the aggregation (in white) and for each service (in grey). Use Case #2 obtained the best scores for quality, with more than 91 % of the services above 5 and a narrow value for the standard deviation (2.54). This means that the quality is very predictable and it is easy to obtain a good quality in a EUD environment. There were only 2 services assessed with a zero in all the analyzed questions.

The Use Case #1 also obtained quite good scores with more than 90 % of the created services above 5, although compared to the Use Case #2 had slightly lower quality scores and a wider standard deviation. In this case the services evaluated with zero were 4.

As shown in Fig. 7 services for the Use Case #3 and #4 obtained lower scores on quality. Only 1 service score above 5 in case of Use Case #3, and 5 in case of Use Case #4. The quality scores were very low for these services because it is the most difficult to build. In an end-user environment, the users can achieve high quality products as long as it is not too difficult for them. Therefore in an end-user environment, it is advisable to empower all the training activities to improve user’s capabilities in order to enhance the quality of the services produced by them.

### Analysis of the discharged patients’ perception of an AmI application

28 discharged patients over 60 years old participated in this study where their perception about the AmI environment was evaluated. They were asked through a personal interview survey about several metrics for the evaluation of AmI environments proposed by Scholtz [19]; in this research work we used a subset of these metrics as shown in Table 2.

**Fig. 8:**
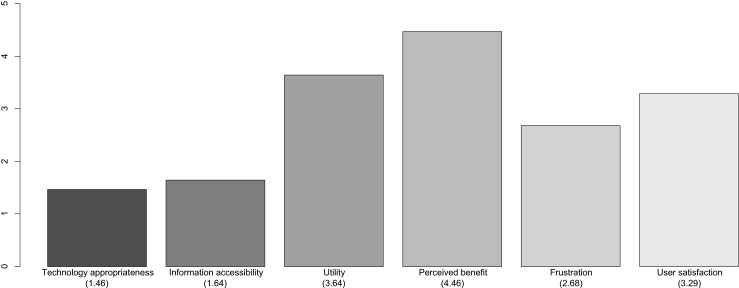
Patients’ perception of an AmI environment

A summary of the assessment made by the patients is shown at Fig. 8. The first metric is the lowest evaluated: “Technology appropriateness”; patients expressed discomfort with the use of mobile devices but not with the mobile application. They did not understand the need to use a smartphone or tablet to receive reminders; in many cases they proposed us to use other ways to receive those reminders such as a phone call. They also have problems obtaining important information provided by the application, as suggested by the low score obtained with the “Information accessibility”.

The frustration level obtained is meaningful and should be lower. Once again the patients express their frustration with the technology and the interaction with the mobile devices.

On the other hand the “Perceived benefit” was the most valued metric (4.46), followed by “Utility” (3.64); meaning that patients are aware of the need to monitor the medicine prescriptions; they understand that a better monitoring of medicines will improve their quality of life. They also valued their satisfaction fairly high (3.29) despite the lower values obtained in “Technology appropriateness” and “Information accessibility” assessments.

This study demonstrates that patients are aware of the usefulness of monitoring the medicines they take, but we must bear in mind that we are dealing with people over 60 years and we must facilitate the interaction with the applications.

### Study of the waiting time reduction when patients receive their medicines

96 persons were observed at the drug delivery stand at the pharmacy; 38 of them carried an RFID card with his/her prescription associated to it (in addition to the classical prescription), and 58 persons only carried the prescription. The experts measured the time since the patient give the prescription to the pharmacist until he/she leaves the stand. The pharmacy staff received a message when the AmI environment detects a patient with an RFID card is on the main entrance; allowing them to prepare the medicines while they are coming to the stand.

**Fig. 9:**
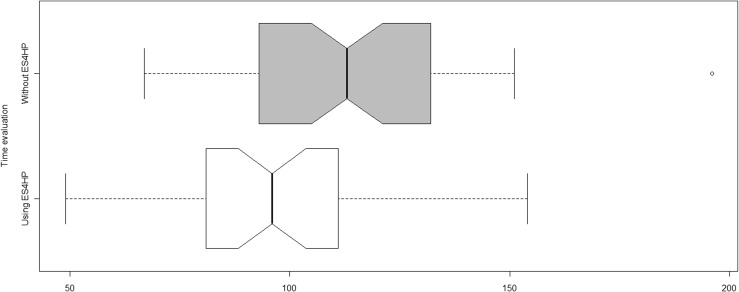
Time measurements’ distributions

Figure 9 shown two boxplot measured in seconds. The grey one shows the distributions of measurements taken to patients with the classical prescription (median = 113s, SD = 26.21), the white boxplot shows the measurements taken to patients who carried the RFID card (median = 96s, SD = 25.21).

A Mann–Whitney *U* test was conducted to confirm whether the use of an AmI environment help to reduce the waiting time at pharmacy delivery stand. The results are positive and support the previous assertion within the expected significance, *p* < 0.005 (U-values omitted as the comparison results have relatively little importance).

## Conclusions

This research work presents an AmI development deployed at the pharmacy department of Gregorio Marañón Hospital for tracing drugs and people at the hospital pharmacy. This research work was designed in order to answer the proposed three research questions that also guide this section:

### Quality of the services created by end-user (the pharmacy specialists)

The statistical study carried out in the experimental validation indicates that the quality of the services in general is good but it depends on the difficulty of the use cases to be solved with the specific service. In this way, End-users can create their own services but always taking into account that the AmI environment must be easy to understand and configure by them.

The pharmacy specialists had no trouble identifying the elements “events” and “actions” of the services they created; however they presented serious problems to model correct messages and many of the services failed the tests. In case of the correct messages, it is mandatory for a EUD AmI application, that all the customizable items are clear and concise enough.

To facilitate the knowledge dissemination among the pharmacy specialists we propose the use of forums and chats to share knowledge, experiences and services as proposed in [20]. In case of the test results, almost no service performed its function as expected when the services were tested by experts. This is because ES4HP Manager does not implement any test environment. It is mandatory for a EUD environment to have a test environment where the users can check by themselves if their services perform the functions expected.

From the statistical study and observations of researchers, we propose several strategies in order to empower the quality of the services: Improve the end-user training; promote their learning and engage them in the AmI environment.

This section can conclude that EUD for AmI environments becomes a reliable solution where users can create their own functionality in a controlled environment, bearing in mind that this functionality must not be very complex to implement; and it is very important to implement a training strategy for users to improve their skills.

### Discharged patients’ perception of an AmI environment application

Results were satisfactory when the patients were asked about their perceived benefit, the utility of the application and their satisfaction. But there are some aspects that need to be improved, for example the technology used: smartphones and tablets. From the interviews with them we can confirm that they were very discouraged by the use of a mobile device, because of its disadvantages (you have to carry it all day, charge the battery very often, need to be an experienced user to know how to use it, it is expensive, fragile, etc.).

They understand the need to monitor the prescribed medicines and they consider it very important but it is necessary to provide another kind of devices easier to handle, wearable, and more enjoyable. Consumer electronics products are aimed to middle aged consumers but it is hard to find electronic products for active aging without being considered geriatric products.

As conclusions for this section we found a technological solution to monitor medicines through ubiquitous applications and to connect the patients with their physician recommendation; but so far we have not found a technological support accepted by users to implement this solution.

### Waiting time reduced when patients receive their medicines

Authors tested whether an application at the pharmacy could save time for patients. They measured 38 people with an RFID card (test group) and 58 people with the classical prescription (control group).

Through statistical analysis it can be concluded that people with RFID card took on average 17 s less to receive their medication.

This is a very simple example of a process that can be improved with an AmI environment, but it shows the potential of these environments and the benefits that can be obtained.
